# *In silico* Identification of Novel Chitinase-Like Proteins in the Silkworm, *Bombyx mori*, Genome

**DOI:** 10.1673/031.012.15001

**Published:** 2012-12-27

**Authors:** Ye Pan, Peng Lü, Yong Wang, Lijing Yin, Hexiang Ma, Guohong Ma, Keping Chen, Yuanqing He

**Affiliations:** ^1^ The Laboratory Animal Research Center, Jiangsu University, No. 301 Xuefu Road, Zhenjiang 212013, P.R China.; ^2^ Institute of Life Sciences, Jiangsu University, 301 Xuefu Road, Zhenjiang 21201 3, P. R. China; ^3^ School of Food and Biological Engineering, Jiangsu University, 301 Xuefu Road, Zhenjiang 21201 3, P. R. China; ^#^ These authors contributed equally to this paper.

**Keywords:** blast search, *Bombyx mori* chitinases, phylogenetic analysis

## Abstract

In insects, chitinases participate in the periodic shedding of old exoskeletons and the turnover of peritrophic membranes. Chitinase family members have been identified in dozens of species, including *Tribolium castaneum, Drosophila melanogaster*, and *Anopheles gambiae*. In this study, nine chitinases and three hypothetical chitinases have been identified in *Bombyx mori* L. (Lepidoptera: Bombycidae) through genome-wide searching. Phylogenetic analyses revealed that seven of them belong to the seven chitinase groups, respectively. BmCht25 and BmCht26 could not be grouped into the known chitinase groups, and might belong to two new groups of the chitinase family. BmCht10, BmCht25, and BmIDGF have glutamate amino acid substitutions in the active catalytic domain. Only BmCht5 and BmCht10 contain CBD domain and PEST sequences (rich in proline, glutamic acid, serine, and threonine). *BmCht5* and *BmCht26* are located on chromosome 7, and others (BmCht6, BmCht7, BmCht10, BmCht11, BmCht20, BmIDGF) are located on separate chromosomes of *Bombyx mori*, respectively. The present study provides important background information for future studies using *Bombyx mori* as a model organism for insect development and virus and host interaction.

## Introduction

Chitin is an insoluble polysaccharide of Nacetylglucosamine, which is a special and important biological polymer of arthropods. It serves as a major structural component of the insect epidermis, trachea, and peritrophic membrane of the intestinal epithelial cuticle. Peritrophic matrix may protect the midgut from infection by pathogens and physical damage by food particles (Shen and Jacobs Lorena 1999). Insects' growth and metamorphosis are strictly dependent on the structural changes of tissues that contain chitin. Coordination of chitin synthesis and its degradation requires strict control of the participating enzymes during development.

Chitinase is an important enzyme responsible for chitin metabolism. Insect chitinases belong to glycoside hydrolase family 18, which has a highly conserved Glyco_18 catalytic domain. Its family members have been found in a wide range of organisms including bacteria, viruses, yeasts, fungi, plants, protozoan parasites, arthropods, and mammals. In humans, six Glyco_18 domain-containing proteins were identified, and the major sources of these proteins are macrophages, neutrophils, epithelial cells, chondrocytes, synovial cells, and cancer cells. Mammalian chitinases are associated with various human disorders and can be used as major and supplementary markers for numerous inflammatory and malignant disorders (Kzhyshkowska et al. 2007). Chitinases have also been identified in the genome of *Autographa californica* nucleopolyhedrovirus (AcNPV) and *Bombyx mori* nucleopolyhedrovirus (BmNPV), both of which belong to the family *Baculoviridae* and attack insects and other arthropods. The baculoviral chitinases may be essential for causing final host liquefaction at the late stage of infection (Merzendorfer H. 2003).

In insects, chitinases have a defensive role when bacteria and fungi penetrate the peritrophic membrane and play a crucial role in larval molting and pupation. *Anopheles gambiae* midgut chitinase seems to act in concert with a chitin synthase to modulate the thickness and permeability of the peritrophic membrane (Shen and Jacobs Lorena 1997). *Ostrinia nubilalis* midgut-specific chitinase plays an important role in regulating chitin content of the peritrophic membrane and subsequently affecting the growth and development of the larvae (Khajuria et al. 2010). Recently, a fat body-specific chitinase was detected in *Glossina morsitans* milk gland tissue and could be important for the development of intrauterine larvae (Merzendorfer H. 2003).

One of the structural features observed in many of these insect chitinases is a multidomain architecture that includes a signal peptide, one or more Glyco_18 catalytic domains, chitin-binding domains (CBD), and a PEST sequence rich in proline, glutamic acid, serine, and threonine. The domestic silkworm, *Bombyx mori* L. (Lepidoptera: Bombycidae), is a central model system for Lepidoptera genomics and genetics, and is also an economically important insect. In this paper, nine *B. mori* chitinases, three hypothetical chitinases identified in the *B. mori* genome database, and an analysis of these sequences are reported.

## Materials and Methods

### tBLASTn searches

*Tribolium castaneum* and *Drosophila melanogaster* chitinases (Zhu et al. 2004; Zhang et al. 2011) were downloaded from GenBank (http://www.ncbi.nlm.nih.gov). and the sequences of Glyco_18 catalytic domain were used to search for similar genes in the silkworm genome sequence with tBLASTn search against database of *B. mori* genome draft sequences (http://www.ncbi.nlm.nih.gov/sutils/genom_ta ble.cgi?organism=insects, and http://silkworm.genomics.org.cn/). The obtained sequences were predicted using FGENESH+ to find the potential chitinase sequences. Determination of the chromosome location of *B. mori* chitinases was carried out with SilkMap program (http://silkworm.swu.edu.en/silksoft/silkmap.html).

### Domain analysis of predicted *B. mori* chitinases

Pfam (http://pfam.wustl.edu/) and SMART (http://smart.embl-heidelberg.de/) domain analysis programs were used to predict the domain architecture of potential chitinases. PEST sequence was predicted by ePESTfind program (http://emboss.bioinformatics.nl/cgibin/emboss/epestfind).

### Sequence alignment

Multiple sequence alignment was performed using the ClustalW program (http://www.ebi.ac.uk/clustalw/). Previous studies have shown four highly conserved signature sequences in the amino acid sequences of all known insect chitinases (Zhang et al. 2011): FDGXDLDWEYP (the residue E is a putative proton donor in the mechanism, and is important for catalytic activity), KXXXXXGGW, MXYDXXG, and GXXXWXXDXD. The sequences that had none of these four conserved sequences were discarded.

### Phylogenetic analysis

Phylogenetic analyses to all the identified *B. mori* chitinase members were carried out in two steps. First, all obtained catalytic domain sequences of *B. mori* chitinases were used to build a neighbor-joining distance tree with the *D. melanogaster* and *T. castaneum* chitinases catalytic domain sequences using MAGA5.0 (Tamura et al. 2011). Then, each catalytic domain sequence of *B. mori* chitinases was used to conduct in-group phylogenetic analyses (Wang et al. 2007). The neighborjoining trees were bootstrapped with 5,000 replicates to provide information about their statistical reliability.

## Results and Discussion

### The chitinase members in *B. mori*

The catalytic domain sequences of chitinases from *D. melanogaster* and *T. castaneum* were used to carry out a tBLASTn search against the *B. mori* genome database. The four conserved regions of catalytic domain amino acid sequences were included in all the reference chitinases used for tBLASTn searching.

**Figure 1.**
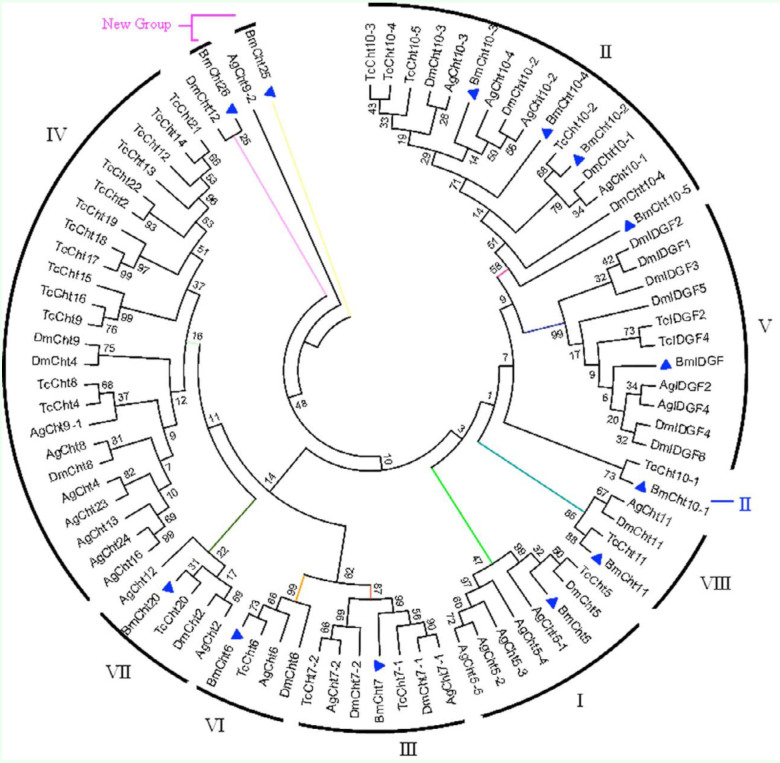
Phylogenetic analysis of *Bombyx mori* and other insect chitinase proteins. A phylogenetic tree was constructed by the neighbor-joining method based on catalytic domain sequences. Sequences used for the analysis are: NP_477298.2 (DmCht2), NP_524962.2(DmCht4), NP_572598.1 (DmCht6), NP_647768.2 (DmCht7), NP_611542.1 (DmCht8), NP_611543.3 (DmCht9), EAA46011.1 (DmCht10), NP_572361.1 (DmCht11), NP_726022.1 (DmCht12), NP_477258.1 (DmIDGF1), NP_477257.2(DmIDGF2), NP_723967.1 (DmlDGF3), NP_727374.1 (DmlDGF4), NP_61121.3 (DmlDGF5), NP_477081.1 (DmlDGF6), NP_00103451 6.3 (TcCht2), NP_001073567.1 (TcCht4), NP_0041034524.1 (TcCht5), XP_967813.1 (TcChto), NP_001036035.1 (TcCht7), NP_001038094.1 (TcCht8), NP_001038096.1 (TcCht9), NP_001036067.1 (TcCht10), XP_974461.1 (TcCht11), XP_972802.2 (TcCht12), NP_001036034.1 (TcCht13), XP_973005.1 (TcCht14), XP_973077.1 (TcCht15), NP_001034515.1 (TcCht16), XP_972719.1 (TcCht17), XP_973161.2 (TcCht18), XP_973119.2 (TcCht19), XP_970191.2(TcCht20), NP_001034517.1 (TcCht21), NP_001038095.1 (TcCht22), NP_001038092.1 (TclDGF2), NP_001038091.1 (TclDGF4), XP_315650.4 (AgCht2), XP_315351.4 (AgCht4), HQ456129 (AgCht5-1), HQ456130 (AgCht5-2), HQ456131 (AgCht5-3), HQ456132 (AgCht5-4), HQ456133 (AgCht5-5), XP_001 687765.2 (AgCht6), XP_308858.4 (AgCht7), XP_316448.2 (AgCht8), XP_307732.4 (AgCht9), XP_001238192.2 (AgCht10), XP_310662.4 (AgCht11), XP_316142.4 9 (AgCht12), XP_3 14312.4 (AgCht 13), XP_3 19801.4 (AgCht16), XP_001 688641.1 (AgCht23), XP_31 6256.4 (AgCht24), XP_001237925.1 (Ag IDGF2), XP_317398.3 (AglDGF4). High quality figures are available online.

**Figure 2.**
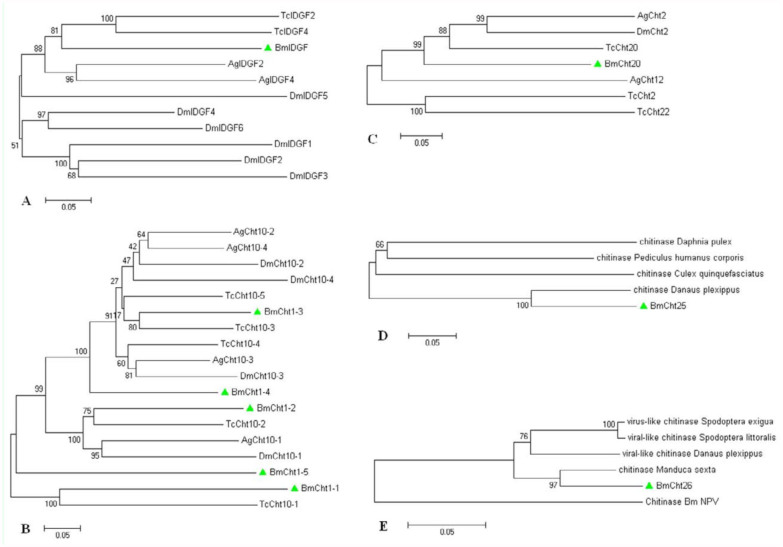
In-group phylogenetic analyses of BmIDGF (A), BmCht10 (B), BmCht20 (C), BmCht25 (D), BmCht26 (E). Neighborjoining trees were constructed with selected group members of the catalytic domains. Sequences used for the analysis are: (A– C) Protein accession numbers are shown in Figure 1. (D) *Culex quinquefasciatus* chitinase XP_00186961 7.1, *Danaus plexippus* chitinase EHJ68452.1, *Daphnia pulex* chitinase EFX69014.1, *Pediculus humanus corporis* chitinase XP_002428336.1. (E) BmNPV chitinase NP_047523.1, *Manduca sexta* chitinase-h ABB88891.1, *Danaus plexippus* viral-like chitinase EHJ71822.1, *Spodoptera littoralis* viral-like chitinase ABA06505.1, *Spodoptera exigua* virus-like chitinase ADI24347.1. High quality figures are available online.

There were nine *B. mori* chitinase proteins identified by tBLASTn searches and sequence analysis. They had four conserved regions of catalytic domain (Table 1). Because the following phylogenetic analyses (Figure 1 and Figure 2) provided support for allocating the identified *B. mori* chitinases into their corresponding families, these chitinases were named as Bm followed by the family name plus a number if more than one member was found in that family. However, two *B. mori* chitinases cannot form monophyletic groups with *T. castaneum, A. gambiae*, or *D. melanogaster* chitinase. These two genes were named as BmCht25 and BmCht26 in accordance with the nomenclature used by Zhang et al (2011). BmCht5 (GenBank accession No.: AAB47538), BmCht26 (GenBank accession No.: BAC67246), and BmIDGF (Imaginai disc growth factor, GenBank accession No.: NP_001036847) were previously reported (Kim et al. 1998; Daimon et al. 2003; Wang et al. 2009). These three proteins were renamed based on the phylogenetic analysis of the catalytic domain. Three *B. mori* hypothetical chitinases protein were found to have incomplete catalytic domain, and these genes were named as *B. mori* hypothetical chitinase protein 1, 2, and 3, respectively.

**Table 1.**
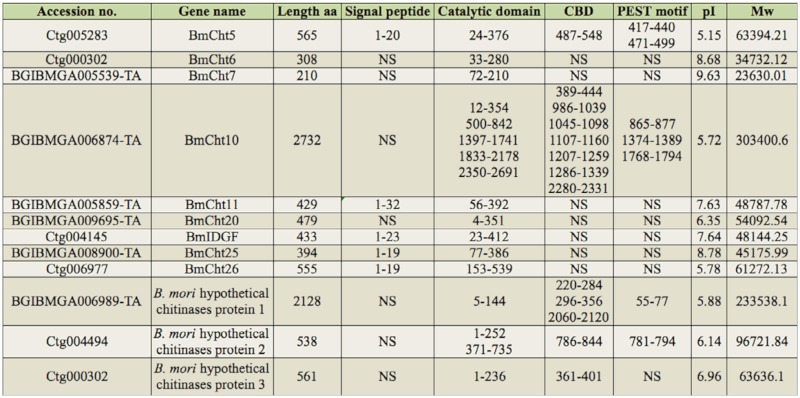
Accession number in NCBI database, amino acid residues, structural domains, pl and Mw values of the predicted *Bombyx mori* chitinases. NS: Not shown in prediction.

Nakabachi et al. (2010) found 12 *B. mori* chitinases when they studied chitinase-like proteins encoded in the genome of *Acyrthosiphon pisum*. Ten of the sequences were the same as were found in the present study (BmCht5, BmCht6, BmCht7, BmCht10, BmCht11, BmCht20, BmIDGF, BmCht25, BmCht26, *B. mori* hypothetical chitinase protein 1), but the other two sequences they mentioned may not belong to glycoside hydrolase family 18, because they do not contain conserved sequences of Glyco_18 catalytic domain as shown by the analysis with SMART tool.

### Identification of orthologous families

Identifying orthologous genes is accompanied with much uncertainty because there is no absolute criterion that can be used to decide if two genes are orthologous (Ledent and Vervoort 2001). In the present study, the criterion used was that a single *B. mori* chitinase must form a monophyletic group with another chitinase of a known family in phylogenetic trees constructed with different methods, and all the bootstrap values must exceed 50 (Zhu et al. 2004; Zhang et al. 2011).

The BmCht5, BmCht6, BmCht7, and BmCht11 formed monophyletic groups with *T. castaneum, A. gambiae*, and *D. melanogaster* catalytic domains, had bootstrap values greater than or equal to 50, and belong to Group I, Group VI, Group III, Group VIII, respectively (Figure 1). Bootstrap values of BmIDGF, BmCht10, BmCht20, BmCht25, and BmCht26 were not greater than or equal to 50. Further phylogenetic analyses showed that BmIDGF belongs to Group V (Figure 2A), BmCht10 belongs to Group II (Figure 2B), and BmCht20 belongs to VII (Figure 2C). BmCht25 and BmCht26 were not included in any of the chitinase groups (Groups I–VIII) (Zhang et al. 2011). They may belong to two new groups of the chitinase family (Figure 2D and 2E). BmCht25 had 78% sequence identity with *Danaus plexippus* chitinase (GenBank accession No. EHJ68452) and 44% identity with *Culex quinquefasciatus* chitinase (GenBank accession No. XP_001869617). BmCht26 had 92% sequence identity with *Manduca sexta* chitinase (GenBank accession No. ABB88891) and 91% identity with *Scania Cynthia* chitinase (GenBank accession No. BAE16586).

### Gene Structure and Chromosome Location of *B. mori Chitinases*

**Figure 3.**
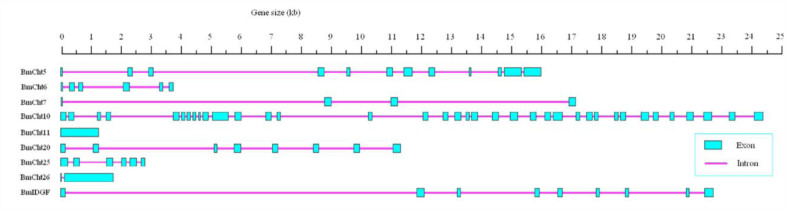
Schematic diagram of exon and intron organization of the chitinase genes from *Bombyx mori*. High quality figures are available online.

**Figure 4.**
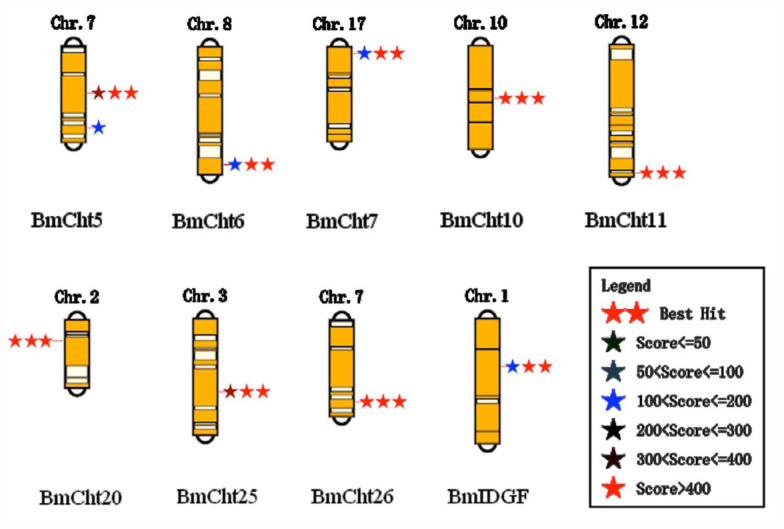
Chromosomal locations of *Bombyx mori* chitinase genes. High quality figures are available online.

BmCht11 has only one exon and BmCht26 has two exons, whereas other *B. mori chitinase* genes have more than two exons (Figure 3). For example, has 9 exons and *BmCht5* has 10 exons (Figure 3). *B. mori* contains 27 autosomes and one Z chromosome. In this study, all identified *B. mori chitinases* genes on the silkworm chromosomes were mapped successfully. *BmCht5* and *BmCht26* are located on chromosome 7. Other *B. mori chitinases* (BmCht6, BmCht7, BmCht10, BmCht11, BmCht20, BmIDGF) are located on separate chromosomes, respectively (Figure 4).

### Domain analysis of *B. mori* chitinases

A multi-domain structural organization is often observed in polysaccharide-degrading enzymes, where one or more domains are responsible for hydrolysis and associating with the solid polysaccharide substrate. Catalysis in family 18 chitinases depends on catalytic domains. CBD domains in the chitinases are presumably to anchor the enzyme tightly onto the substrate. The linker region apparently helps to stabilize the enzyme and protects protease-susceptible bonds in the catalytic domain from hydrolysis in the gut. The PEST sequence in the linker region serves as proteolytic signals. With such domains, insect chitinases may evolve from efficient degradation of the insoluble polysaccharide to soluble oligosaccharides during the molting process (Kramer 1997; Arakane et al. 2003).

**Figure 5.**
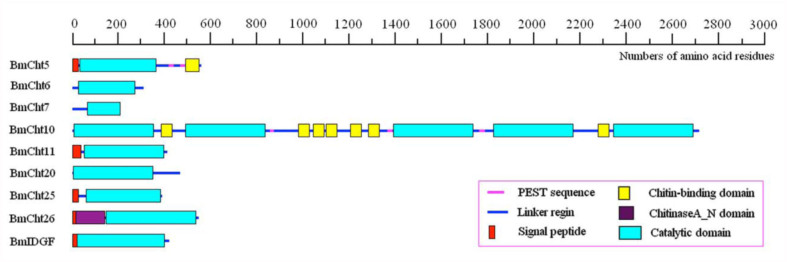
Domain architecture of identified *Bombyx mori* chitinase proteins. All the sequences contain at least one catalytic domain. The programs Pfam and SMART were used to analyze the identified sequences. High quality figures are available online.

**Figure 6.**
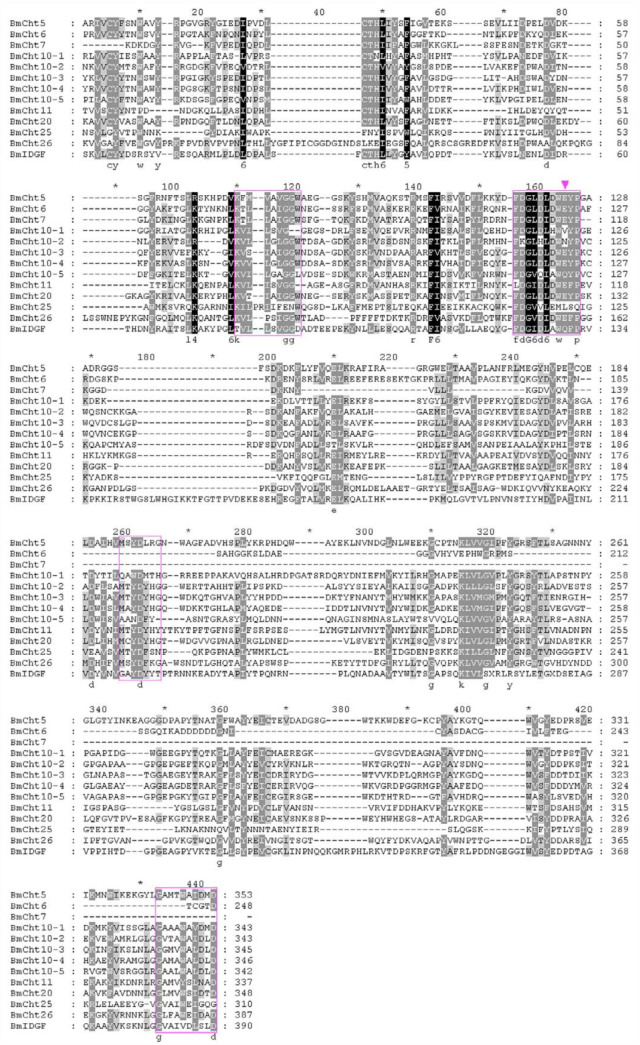
Multiple sequence alignment of the catalytic domains of *Bombyx mori* chitinase proteins. Catalytic domain sequences were retrieved from the identified *B. mori* chitinase proteins and the ClustalW program was used. The four conserved regions are boxed. The arrowhead indicates the important glutamate amino acid. High quality figures are available online.

Domain analysis of the nine *B. mori* chitinases showed that they contain at least one Glyco_18 catalytic domain (Figure 5). The Glyco_18 catalytic domain with FDGXDLDWEYP sequence is predicted to be active catalytically, and the glutamate amino acid (E) substitutions in these sequences are predicted to be non-enzymatic. Most likely, these proteins are lectins that will bind to chitin or other carbohydrates containing Nacetylglucosamine. They may be involved in cell-to-cell communication or in insect immunity. BmCht10 has five catalytic domains. The first catalytic domain has a glutamate-valine (E-V), the second catalytic domain has a glutamate-asparagine (E-N), the fifth catalytic domain has a glutamateGlutamine (E-Q), and the other two catalytic domains retain glutamate residues. Both BmIDGF and BmCht25 contain one catalytic domain, which have glutamate-glutamine (EQ) and glutamate- leucine (E-L), respectively (Figure 6).

**Figure 7.**
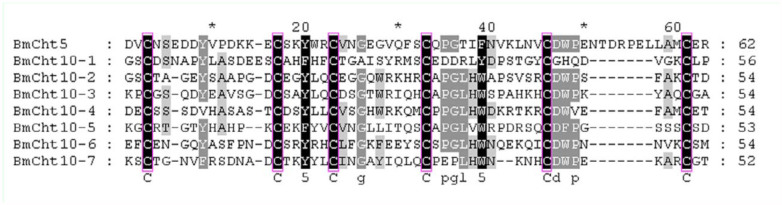
Multiple sequence alignment of CBD domains of *Bombyx mori* chitinase proteins. CBD domain sequences were retrieved from the identified *B. mori* chitinases and analyzed using the ClustalW program. The six cysteine residues are conserved among the chitin-binding domains of *B. mori* chitinases. High quality figures are available online.

The function of the CBD domain in chitinases is presumably to anchor the enzyme tightly onto its substrate, thereby facilitating the hydrolytic process. In insect chitinases, the CBD domain has six conserved cysteines that probably form three disulphide bridges (Suetake et al. 2000; Behr and Hoch 2005). BmCht10 contains seven CBD domains, BmCht5 contains one CBD domain, and the other seven *B. mori* chitinases (BmCht6, BmCht7, BmCht11, BmCht20, BmCht25, BmCht26, and BmIDGF) do not contain a CBD domain (Figure 7).

The N-terminal sequence of BmCht5, BmCht11, BmCht25, BmCht26, and BmIDGF contains a highly hydrophobic amino acid that is likely to function as a signal peptide (Figure 3), which means that these chitinases are secreted. Some chitinases contain the sequences rich in proline (P), glutamic acid (E), serine (S), and threonine (T), which qualifies it as a PEST sequence (Rechsteiner and Rogers 1996). The linker region of *Manduca sexta* and *T. castaneum* chitinases also contain potential PEST sequences. It serves as proteolytic signals, necessary and sufficient for ubiquitination (Roth and Davis 2000). BmCht10 contains five catalytic domains that are separated by three potential PEST. It is speculated that BmCht10 may be expressed as zymogens that are subsequently cleaved by proteolysis to reveal multiple active enzymes. BmCht5 also contains two potential PEST sequences. This composition may promote the chitinase precursor to be converted into active form, or suggest that the insect chitinases might be rapidly degraded proteins.

### Function of some *B. mori* chitinases

BmIDGF shows high sequence similarity to chitinase and belongs to group V of the chitinase-like family (Zhu et al. 2008). IDGF is a soluble polypeptide growth factor that was first identified from the conditioned medium of *D. melanogaster* imaginai disc C1.8+ cells. It is also expressed in larval glands and fat body. It is secreted and transported to target tissues via the hemolymph (Kawamura et al. 1999). However, the *D. melanogaster* IDGF, *D. melanogaster* DS47, and *T. castaneum* IDGF were demonstrated to be devoid of chitinolytic activity, although they could act as carbohydrate-binding proteins and bind very tightly to an insoluble ligand-colloidal chitin. In *Mamestra brassicae*, recombinant *M. brassicae* IDGF could stimulate the growth of cells derived from the fat body and hemocytes without insulin (Zhang et al. 2006). Previous work (Wang et al. 2009; Pan et al. 2010) found that BmIDGF was expressed in all developmental stages of silkworm larvae and various larval tissues and was located in the extracellular space. It was predicted that BmIDGF is produced mainly by the fat body, secreted into the hemolymph, and then brought to other organs.

BmCht5 generates four mRNA products (GenBank accession No. NP_001166831, NP_001037480, NP_001166832, and NP_001166833) by alternative splicing. Furthermore, the four mRNA products showed chitinase activity when expressed in *Escherichia coli*, which demonstrates the role of the alternative splicing process in generating multiple isoforms of the silkworm's chitinases. The presence of this splicing mechanism in the *B. mori* chitinases also contributes to the interpretation of the variations reported in studies of the insect's cDNAs. All of the mRNA products are from a single gene and functionally active (Babiker et al. 2002). Mikitani et al. (2000) found a novel DNA type transposon in BmCht5 that shows similarity to the TC-like transposable element.

BmCht26 showed extensive homology with chitinase of bacteria and baculoviruses. BmCht26 showed 62% sequence identity with BmNPV ChiA and 72% with *Serratia marcescens* chiA. It was suggested that BmCht26 may be derived from a bacterial or baculovirus chitinase gene via horizontal gene transfer (Daimon et al. 2003, 2005, 2006). The insect chitinases have been found to be endochitinases. In a recent study, BmCht26 had exo-type substrate preference just as baculoviruses ChiA (Daimon and Katsuma 2007), which is different from other *B. mori* chitinases. BmCht26 localizes in the chitincontaining tissues during the molting stages, indicating that it plays a role in chitin degradation during molting.

Interestingly, *B. mori* hypothetical chitinase proteins 1, 2, *and* 3 were found to have an incomplete catalytic domain that had lost the FDGXDLDWEYP and KXXXXXGGW conserved regions, but all of them retained the CBD domain. They may be hypothetical chitinase proteins in the silkworm genome (Table 1). They cannot form monophyletic groups with *T. castaneum, A. gambiae*, or *D. melanogaster* chitinases (data not shown). Nevertheless, it was found that *T. castaneum, Danaus plexippus*, and *Pediculus humanus corporis* also have a number of hypothetical chitinase sequences (GenBank accession No. EFA05323, EHJ77971 and XP_002425481), all of which have high sequence identity with the three *B. mori* hypothetical chitinase proteins. Whether they are genuine chitinase proteins and have chitinase-like functions remains to be determined by further studies.

In *D. melanogaster*, sixteen chitinases were identified. In *T. castaneum*, twenty-two chitinases were identified. In *A. gambiae*, twenty chitinases were identified (Zhang et al. 2011). In this work, nine *B. mori* chitinases and three hypothetical chitinase proteins were identified in the current version of silkworm genome sequences. Therefore, identification of more *B. mori* chitinases is expected upon release of a new version of the silkworm genome. Protein expression and chitinase activity assays, as well as chitin-binding studies, are needed to verify that the sequences identified in this study are indeed *B. mori* chitinase proteins.

## Abbreviations

Ag,: *A. gambiae;*
Bm,: *B. mori*;
CBD,: chitin-binding domains;
Dm,: *D. melanogaster;*
IDGF,: imaginai disc growth factor;
Tc,: *T. castaneum*
